# Oxymetholone-Induced Acute Renal Failure: A Case Report

**DOI:** 10.22088/cjim.9.4.406

**Published:** 2018

**Authors:** Azade Tarashande foumani, Forouzan Elyasi

**Affiliations:** 1 Nasibeh Nursing and Midwifery School, Mazandaran University of Medical Sciences, Sari, Iran; 2 Psychiatry and Behavioral Sciences Research Center, Addiction Institute, Comprehensive cancer center, School of Medicine, Mazandaran University of Medical Sciences, Sari, Iran.

**Keywords:** Anabolic steroids, Oxymetholone, Rhabdomyolysis, Renal failure

## Abstract

**Background:**

The prevalence of using anabolic steroids such as oxymetholone is increasing. This highlights the need for closely monitoring side effects of this drug. Acute renal failure (ARF) has been reported as a complication of rhabdomyolysis in anabolic steroids users.

**Case presentation:**

We present one 33-year-old man complaining of decreased urine volume, urine color change, and lower abdominal pain. He is engaged with a rare side effect of oxymetholone abuse. During assessments of potential medical issues associated with the intake of anabolic steroids, known side effects are known to be transient, but the need for appropriate interventions remains essential.

**Conclusions:**

Rhabdomyolysis due to drug use and the consequent acute kidney injury are among the lethal risks associated with anabolic steroid abuse. In most cases, the symptoms are extensive and often misleading. Therefore, detailed history taking, physical scrutiny, paraclinical testing, and early diagnosis are crucial for rhabdomyolysis patients.

Following the isolation and identification of testosterone in 1935, attempts were made to synthesize numerous corresponding derivatives (1, 2). Testosterone is characterized on the basis of its effects, and testosterone derivatives are known as androgenic-anabolic steroids (AASs) because of their functions. Some of the androgenic effects of testosterone include the growth of male reproductive system and the development of secondary sexual characteristics (3, 4). Its anabolic effects include the stimulation of protein synthesis, creation of positive balance between nitrogen concentration and muscle growth, enhancement of calcium absorption, stimulation of skeletal growth, reduction of body fat, increasing electrolyte uptake, and hematopoiesis (3). AASs are also used to treat compensated hypogonadism, catabolic disorders, muscular dystrophy, growth retardation, tissue repair, osteoporosis, aplastic anemia, and breast carcinoma (3, 5). They can be administered in both oral and injectable forms, such as oxandrolone, oxymetholone, methandrostenolone, stanozolol, nandrolone, and decanoate (6, 7). The above-mentioned properties of AASs has once motivated professional bodybuilders to take these substances, but their use has been gradually spreading among other people (1). Most athletes use AASs to achieve a trained physique and increase their muscle size, whereas others often employ the substances to enhance libido and augment muscle mass (3). According to international reports, the prevalence of AAS use is 3% to 4% among men and 1.6% among women (1).

The increasing prevalence of AAS use highlights the need for closely monitoring their side effects, such as decreased secretion of sex hormones, testicular atrophy, oligoazoospermia, impotence, prostatic hypertrophy, gynecomastia in men, menstrual irregularities, clitoral hypertrophy, urethral atrophy, atrophy of the female breast, liver cell damage, cholestasis, hepatoadenoma, cholesterol elevation, hypertension, thrombosis, reduced glucose tolerance, acne, alopecia, male pattern baldness, edema, hirsutism, and hoarseness (6, 8-10).

Acute renal failure (ARF) has been reported as a complication of rhabdomyolysis in AAS users (1). Rhabdomyolysis is defined as the breakdown of muscle tissue through the dispersion of intracellular contents into extracellular fluid (11). Corresponding clinical consequences vary from muscle weakness to life-threatening ARF (12), and rhabdomyolysis severity and degree can be diagnosed clinically by increasing the concentration of creatinine phosphokinase (CPK) (13) and lactate dehydrogenase (LDH) (10). A patient with symptoms such as myocyte edema, increased intravascular volume, and urine color change to reddish-brown is admitted for the clinical diagnosis (13). Today, rhabdomyolysis causes ARF in rare cases only (13). Rhabdomyolysis occurs for various reasons, including poisoning, ischemia, infection and inflammation, severe metabolism, and extreme physical activity (10), but it can also develop after the intake of certain medications, such as steroids; although its incidence is rare, the syndrome is a life-threatening phenomenon (11). The renal side effects of AASs are infrequent and have been documented only in a few cases within some separate reports (14). The next section discusses a patient who got engaged with one of the rare side effects of anabolic steroids due to oxymetholone consumption. 

## Case presentation

The reported patient was a 33-year-old man complaining of decreased urine volume, urine color change, and lower abdominal pain. He was admitted to the emergency department of a general university hospital in one of the northern cities of Iran in winter, 2016. After preliminary examinations, he was transferred to the department of nephrology for dialysis and other treatments because of high levels of urea and creatinine as well as ARF. Because of a history of drug dependence, a request for psychiatric consultation was submitted to psychiatric service department of the hospital. During clinical diagnostic interviews, the patient was determined as suffering from substance-related disorder and borderline personality disorder on the basis of the Axes I and II dimensions, respectively, of the Diagnostic and Statistical Manual of Mental Disorders, Fifth Edition (DSM-5). 

He had a history of hospitalization for methamphetamine rehabilitation, after which the patient turned to methadone, tramadol, and opium. To prevent the weight loss caused by these drugs, he arbitrarily used oxymetholone for two months. The patient was observed for the onset of urinary symptoms for four days prior to admission. The first laboratory findings for ARF included a urea level of 238 mg/dL and a creatinine level of 11 mg/dL. Muscle injury and rhabdomyolysis were confirmed through the analysis of experimental data (myoglobinuria, CPK: 10726 IU/L and LDH: 4383 U/L). Concentrations of electrolytes, such as potassium and sodium (Na: 130 mEq/L and K: 4.9 mEq/L), and serum levels of liver enzymes (alanine transaminase and aspartate transaminase) and coagulation factors were normal. Liver and kidney ultrasonography was performed shortly after hospitalization. 

Liver size and echogenicity were normal. The right kidney was 136 mm in size, with a cortical echo and increased corticomedullary differentiation. A small amount of perinephric fluid was evident around the kidney. The left kidney was 102 mm in size, with a normal paranshyal echo and reduced focal thickness. Based upon physical examination of the patient, the heart, lungs, and nervous system showed normal functioning. After diagnostic confirmation, the primary therapeutic purpose was to prevent ARF risk factors, including the reduction of fluid volume, the blocking of tubules, release of free radicals, and aciduria. The patient underwent seven rounds of hemodialysis and hydration. Eight days after the first day of hospitalization, his CPK and LDH levels declined rapidly (CPK: 365 IU/L and LDH: 855 U/L). When ARF was settled and the values were normal, the patient was discharged.

## Discussion

According to the laboratory and clinical symptoms, the differential diagnoses for the patient included polymyositis, dermatomyositis, mild muscle injury, infection, compartment syndrome, rhabdomyolysis, and sickle cell crisis (10). First, the presence of the classic triad of rhabdomyolysis, namely, muscle damage, colored urine, and kidney dysfunction, was considered in the diagnosis; the diagnosis of ARF caused by intoxication with tramadol, methadone, and opium was performed. Comprehensiveness of the psychiatric history taking and other available details enabled the clinicians to determine a history of oxymetholone intake by the patient. Nevertheless, the intake of oxymetholone simultaneously with methadone, opium, and tramadol did not cause rhabdomyolysis—a determination made on the basis of the fact that this syndrome involves the breakdown of skeletal myocytes, resulting in the release of intracellular contents into interstitial fluid and the bloodstream (12). As expected, an increase in muscle atrophy-related factors, such as CPK and LDH, in the blood was observed (13). In the reported case, the absence of fever and leukocytosis negated infection are as one of the causes of rhabdomyolysis. Inflammation was also rejected because of the lack of increased inflammatory markers in the patient’s clinical tests.

In conclusion During assessments of potential medical issues associated with the intake of anabolic steroids, the known side effects are known to be transient, but the need for appropriate interventions remains essential. Evidence suggests that, other influencing factors, such as diet and other medications, also largely contribute to serious side effects of anabolic steroid intake. Various factors result in the development of rhabdomyolysis. Drug-resulted rhabdomyolysis and consequent acute kidney injury are among the lethal risks associated with anabolic steroid abuse; these have remained as mostly unknown and obscure risk factors. 

Rhabdomyolysis goes through a very complex mechanism that occurs through many compounds; in most cases, symptoms are extensive and often misleading. Therefore, detailed history taking, physical scrutiny, paraclinical testing, and early diagnosis are crucial for rhabdomyolysis patients. 
